# BioPARR: A software system for estimating the rupture potential index for abdominal aortic aneurysms

**DOI:** 10.1038/s41598-017-04699-1

**Published:** 2017-07-05

**Authors:** Grand Roman Joldes, Karol Miller, Adam Wittek, Rachael O. Forsythe, David E. Newby, Barry J. Doyle

**Affiliations:** 1 Intelligent Systems for Medicine Laboratory, The University of Western Australia, 35 Stirling Highway, Perth, WA 6009 Australia; 2 School of Mechanical and Chemical Engineering, The University of Western Australia, 35 Stirling Highway, Perth, WA 6009 Australia; 3 School of Engineering and Information Technology, Murdoch University, 90 South St, Murdoch, WA 6150 Australia; 4 School of Engineering, Cardiff University, The Parade, CF24 3AA Cardiff UK; 5 BHF Centre for Cardiovascular Science, The University of Edinburgh, Edinburgh, UK; 6 Vascular Engineering Laboratory, Harry Perkins Institute of Medical Research, QEII Medical Centre, and Centre for Medical Research, The University of Western Australia, Perth, WA 6009 Australia

## Abstract

An abdominal aortic aneurysm (AAA) is a permanent and irreversible dilation of the lower region of the aorta. It is a symptomless condition that, if left untreated, can expand until rupture. Despite ongoing efforts, an efficient tool for accurate estimation of AAA rupture risk is still not available. Furthermore, a lack of standardisation across current approaches and specific obstacles within computational workflows limit the translation of existing methods to the clinic. This paper presents BioPARR (Biomechanics based Prediction of Aneurysm Rupture Risk), a software system to facilitate the analysis of AAA using a finite element analysis based approach. Except semi-automatic segmentation of the AAA and intraluminal thrombus (ILT) from medical images, the entire analysis is performed automatically. The system is modular and easily expandable, allows the extraction of information from images of different modalities (e.g. CT and MRI) and the simulation of different modelling scenarios (e.g. with/without thrombus). The software uses contemporary methods that eliminate the need for patient-specific material properties, overcoming perhaps the key limitation to all previous patient-specific analysis methods. The software system is robust, free, and will allow researchers to perform comparative evaluation of AAA using a standardised approach. We report preliminary data from 48 cases.

## Introduction

There are many limitations to the current clinical definition of ‘high-risk’ of rupture for AAA, based mainly on the maximum diameter of the AAA. Many researchers across both engineering and medical disciplines believe that biomechanics based patient-specific modelling (PSM) could have major clinical potential to provide more accurate patient-specific rupture risk assessment^1–4^.

With the advances in medical imaging technology and medical image analysis software, it became possible to create anatomically-correct reconstructions of the AAA, which were then used for computer simulations that have steadily increased in complexity^3, 5–7^. These simulations can compute the stress in the AAA wall due to the internal blood pressure. Mechanically-speaking, rupture of an artery occurs when the local wall stress exceeds the local wall strength. Vande Geest *et al*. proposed a useful statistical model for the non-invasive estimation of AAA wall strength^8^ and also introduced the rupture potential index (RPI)^2^. The RPI combines the estimated patient-specific AAA wall strength with the AAA wall stress computed using the finite element method. The RPI has since been implemented in several AAA rupture risk assessment studies^3, 4, 9–11^ and also in a commercial software for AAA analysis (VASCOPS)^12^.

The current approach to RPI computation, including that implemented in the VASCOPS software, uses routinely acquired computed tomography (CT) data of the AAA to create three-dimensional (3D) reconstructions of the aneurysm. Despite some researchers developing algorithms and methods to measure AAA wall thickness from CT^7, 13, 14^, the poor soft tissue contrast of CT data compared to magnetic resonance imaging (MRI) limits the visibility of the AAA wall. Therefore, the vast majority of previous computational studies on AAA rupture risk assume a uniform wall thickness. A uniform wall is anatomically incorrect and results in inaccurate wall stress distributions, and thus RPI estimates. Therefore, RPI data based on uniform aortic wall thickness could be misleading in a clinical setting. Irrespective of uniform or variable wall thickness methods, the 3D reconstructed geometry is converted into a computational mesh of elements, typically tetrahedral elements due to the ease of automated mesh generation. Material properties must then be assigned to the model and are typically based on population-mean mechanical test data for the AAA wall tissue and the intraluminal thrombus (ILT)^15, 16^. A key obstacle in the translation of RPI to the clinic is the use of average material data, as in doing so, the model deviates away from a patient-specific simulation.

However, in some problems of biomechanics (and mechanics in general), material properties have negligible impact on wall stress^17, 18^. This occurs when the geometry to be analysed is already deformed, as is the case when examining arteries reconstructed from medical images. The medical image data represents the geometry under pressurisation from blood. Much effort has focussed on determining the pressure-free geometry using inverse procedures^17, 19^; however, what was observed when using the inverse method is that wall stress is almost independent of material properties. Building on from this, we have reformulated the mechanics of the AAA problem and demonstrated that AAA wall stress can be computed without knowledge of material properties and through a direct linear analysis^19^.

In this paper, we present our framework and describe the key algorithms and techniques that we have implemented. An important aspect of our software is automation. Besides the semi-automatic 3D reconstruction of the AAA, the analysis is completely ‘push button’, thus eliminating potential inter-user variation and creating a standardised approach.

## Results and Discussion

**Figure 1:**
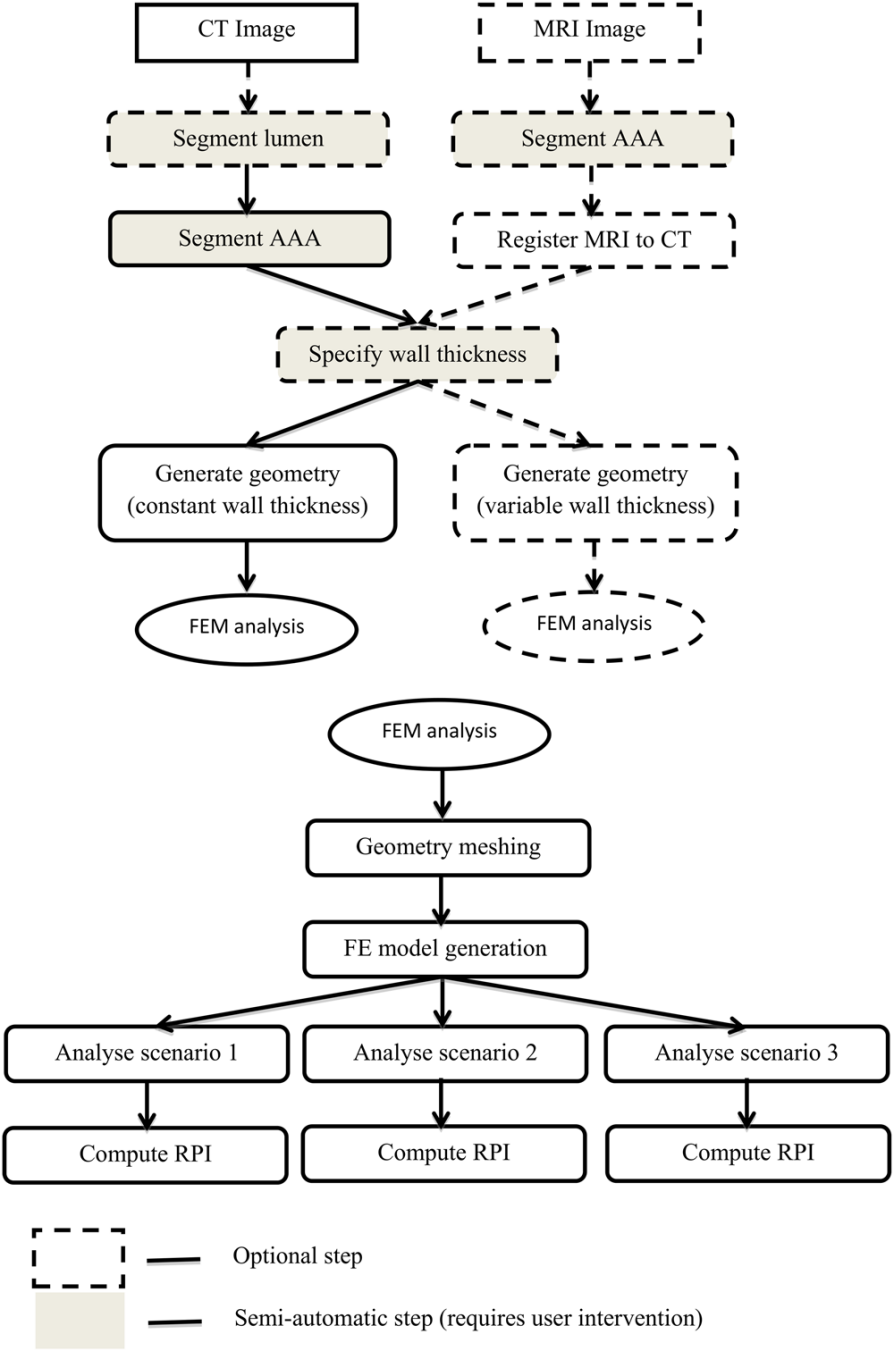
Diagram of the workflow. Only segmentation and wall thickness specification require user intervention; all other steps are automated. Multiple modelling scenarios are analysed (Scenario 1: pressure load on internal ILT wall; Scenario 2: no ILT included; scenario 3: ILT included and pressure load on internal AAA wall). MRI = magnetic resonance imaging, CT = computed tomography, FEM = finite element method.

We have developed a semi-automatic, modular and easily extendable software system for analyzing the rupture potential of an AAA using a finite element analysis based rupture index. The software system, available for free download and usage^20^, consists of a collection of programs and scripts which perform the required steps in the AAA workflow, from image segmentation to geometry creation, meshing, finite element analysis and rupture potential index (RPI) computation (Fig. 1). The software is divided into several modules which perform specific tasks and are being run in sequence from a master script (batch) file. Data communication between these modules is performed using files in standard formats; this allows the user flexibility in changing or extending the functionality of the software, by replacing or adding additional modules.

To guarantee the accuracy of the results, the analysis by the finite element method (FEM) is conducted using the commercial finite element software Abaqus^21^. All the other modules consist of free or open source software programs. The software runs on 64Bit Windows operating systems and has been tested on Windows 7 and 8. We have created a series of tutorial training videos for each step of the analysis; the access link for these resources is included with the software.

Figure 1 shows the developed workflow. The software system allows the analyst to extract and combine data from images of different modality (such as CT and MRI), by using an inter-modality image registration algorithm. The analyst has control over many parameters influencing the analysis results: the thickness of the AAA wall, inclusion of thrombus, geometry meshing, finite element type selection, and finite element simulation scenarios. The software can be used in the case when both CT and MRI data are available for a patient or, the more typical situation, when only CT is acquired.

We have automated the finite element analysis and so have removed the need for technical expertise in computational mechanics. There is still the need to semi-automatically reconstruct the geometry, as this part of the workflow cannot be fully automated. However, this step takes approximately 45 minutes per case and, as with most manual tasks, the required time reduces with increasing familiarity. This segmentation time is comparable with that needed to segment AAA using the commercial VASCOPS software (~40 minutes)^22^. In the future, the segmentation time may be reduced by using better quality images (having enhanced contrast between AAA and the surrounding structures) or through the adoption of emerging image analysis techniques, such as those relying on machine learning^23^. An important aspect of our software is the fast return of data. Our computational approach removes non-linearities (both material and geometric) from the model. As such, the computation time is dramatically reduced and can fit into the clinical workflow.

**Figure 2:**
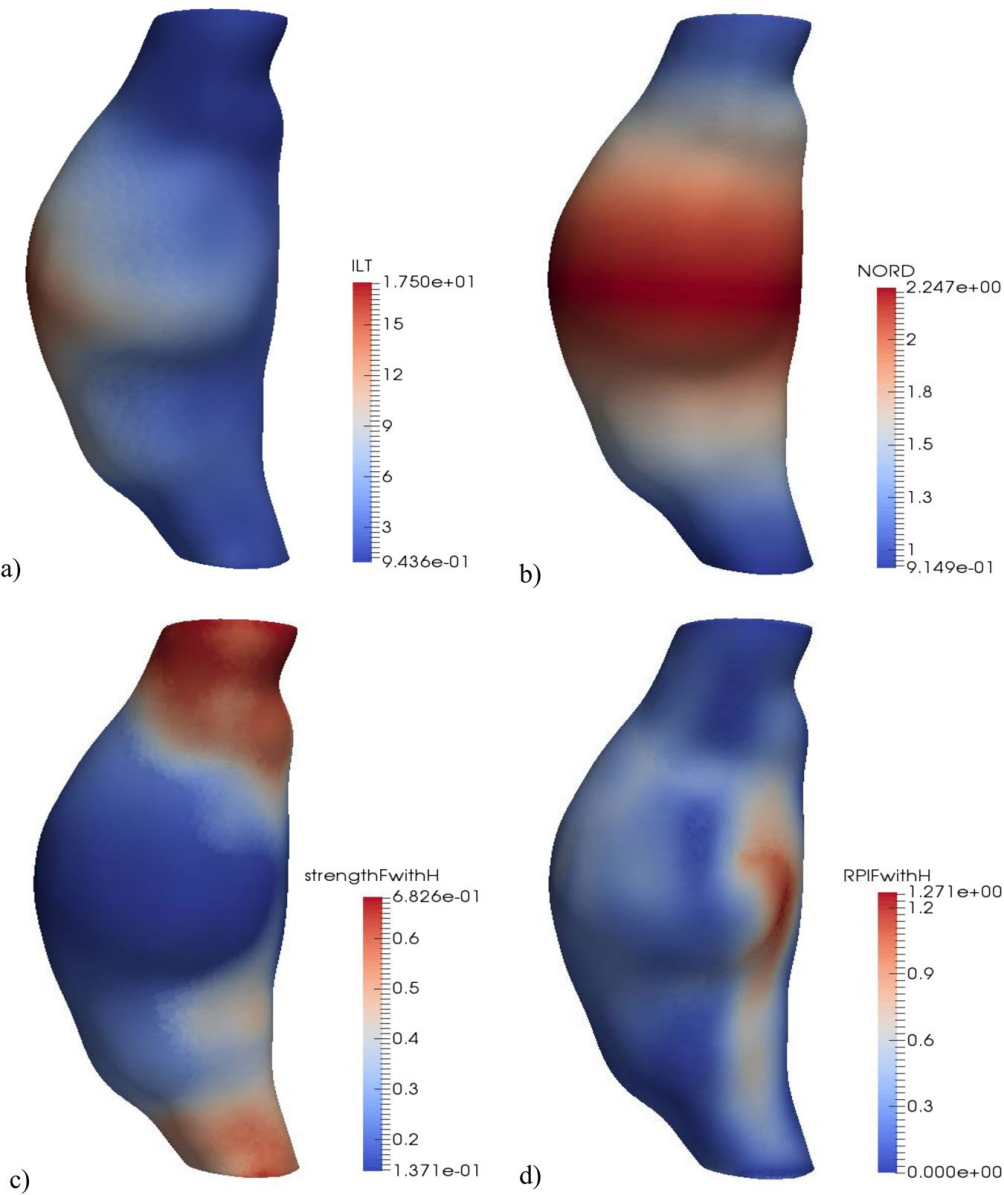
Example of RPI computation results. **(a**) ILT thickness [mm]. (**b**) Normalized diameter (NORD). (**c**) Wall strength for a female with family history of AAA [MPa]. (**d**) RPI for a female with family history of AAA.

Example results generated by our software are presented in Fig. 2. The program automatically generates 3D color-contoured visualizations of the key patient-specific components of the analysis, namely, ILT thickness (ILT), the normalized ratio of the maximum AAA diameter and the diameter in the proximal neck of the aneurysm (NORD), the estimated wall strength, and the final RPI. In this particular example, the patient was female with a family history of AAA; both gender and family history of the disease significantly affect the wall strength estimation. Excluding the 3D geometry reconstruction time, the entire analysis of this scenario took approx. 6 minutes on an Intel(R) Core(TM) i7-5930K CPU @ 3.50 GHz with 64 GB of RAM running Windows 8 OS. The analysis time greatly depends on the problem size (i.e. size of the computational grid, in this case just under 200 k nodes and 740 k elements, and the type of elements used, in this case linear tetrahedrons) and the number of scenarios considered.

In addition to its clinical potential, our software system can be used to answer research questions related to the evaluation of AAA rupture potential index, such as:

- What is the influence of wall thickness and boundary conditions on the computed stress and RPI?
- How many layers of finite elements and what type of elements should be used in the discretization to obtain convergence for the finite element analysis results?
- What simulation scenario leads to the most accurate prediction of stress distribution?
- What combination of simulation parameters leads to the most efficient and accurate RPI?

Finally, a major hurdle to translation of computational biomechanics methods to a clinical setting is robust statistical evidence. Until now, computational methods required intensive analyst time and expertise, and generally resulted in studies that lack statistical power. This problem is further compounded by the lack of standardization of computational methods^24^, which makes meta-analyses of studies difficult^25^. Without statistical evidence that the RPI closely correlates with the rupture risk, clinical uptake is unlikely. Therefore, we have made our software freely available to all^20^, easily expandable and modifiable, and we hope this will enable multi-centre studies of large cohorts to generate statistical evidence.

In conclusion, we have created and offered a free software system for estimating the risk of rupture in abdominal aortic aneurysms. All analysis steps have been automated, except image segmentation, which is impossible to perform automatically given the current image acquisition techniques and image processing algorithms. Therefore, once the software system has been configured, it can be run automatically for different segmented AAA cases and by different analysts without any user intervention; this reduces the analysis time, does not require technical knowledge from the user and generates reproducible results. Having a modular structure with data transfer between modules using standard file formats, the software system is easy to modify and expand.

We used our software system to analyse 48 cases acquired in the initial phase of the MRI in AAA to predict Rupture or Surgery (MA^3^RS) study^26^ – a multicentre observational cohort study of patients under surveillance for AAA with maximum diameter greater than 4 cm (EudraCT 2012-002488-25). It proved reliable and able to handle all cases, which included a large range of AAA sizes and shapes. Once follow-up data of the entire MA3RS cohort is available, we plan to analyse that data and collect the much needed statistical evidence regarding the accuracy of this AAA rupture risk analysis method. Some preliminary results from this analysis are presented in the following subsections, using the assumption of constant AAA wall thickness and a standard blood pressure of 120 mmHg. We used second order tetrahedral elements, for accurate stress computation. The stresses computed near the top and bottom AAA boundaries (which are constrained) were excluded when extracting the maximum stresses or RPI, as stress concentrations occur in these areas.

### Reproducibility of analysis results

Because the entire workflow after image segmentation is automated, the analysis following image segmentation is completely reproducible. Image segmentation involves manual user intervention and its reproducibility depends on the experience of the user and the quality of the images. We have performed the following study to quantify the effect of image segmentation on the computation results:

- Three users with different levels of experience in AAA segmentation have each segmented the AAA and lumen from three CT datasets;
- The segmentations were compared using the Dice Similarity Coefficient (DSC);
- The segmentations were used as input to the analysis framework and the results compared;

The three users are marked as user A (experienced, with many hours of AAA segmentation and usage of 3D Slicer), user B (less experienced, with only a couple of AAA segmentations done using 3D Slicer) and user C (novice, no previous AAA segmentations). Users B and C were instructed by user A in the application of the software and given access to several online tutorials. The results obtained by user A were considered as the best and the results from the other users were compared against them.

The results of the experiment are presented in Table 1. From the data we can draw the following conclusions:

- The accuracy of segmentation increases with the user experience, as indicated by the DSC coefficients;
- Parts of the image with good contrast were easily segmented by all users (there is much smaller difference in the lumen DSC and maximum ILT thickness between users);
- For the novice user, the differences in the computed maximum principal stress were less than 25%.

**Table 1:** Reproducibility of analysis results.

Case	Max. diameter [mm]	User	AAA DSC	Lumen DSC	Max. ILT thickness [mm]	Max. NORD	Max. principal stress [MPa]
1	48	A	1	1	19.8	2.2	0.69
		B	0.982	0.997	20.3 (3%)	2.3 (5%)	0.62 (10%)
		C	0.953	0.981	19.3 (3%)	2.5 (14%)	0.52 (24%)
2	54	A	1	1	19.6	1.8	1.05
		B	0.952	0.983	19.6 (0%)	2.1 (17%)	1.0 (5%)
		C	0.896	0.969	21 (7%)	1.6 (11%)	1.1 (5%)
3	48	A	1	1	16.4	1.8	0.62
		B	0.955	0.961	16.1 (2%)	1.8 (0%)	0.58 (6%)
		C	0.899	0.908	17.3 (5%)	1.7 (6%)	0.75 (21%)

The relative differences between the results of users B and C and user A are shown in parenthesis. NORD = normalized diameter.

**Figure 3:**
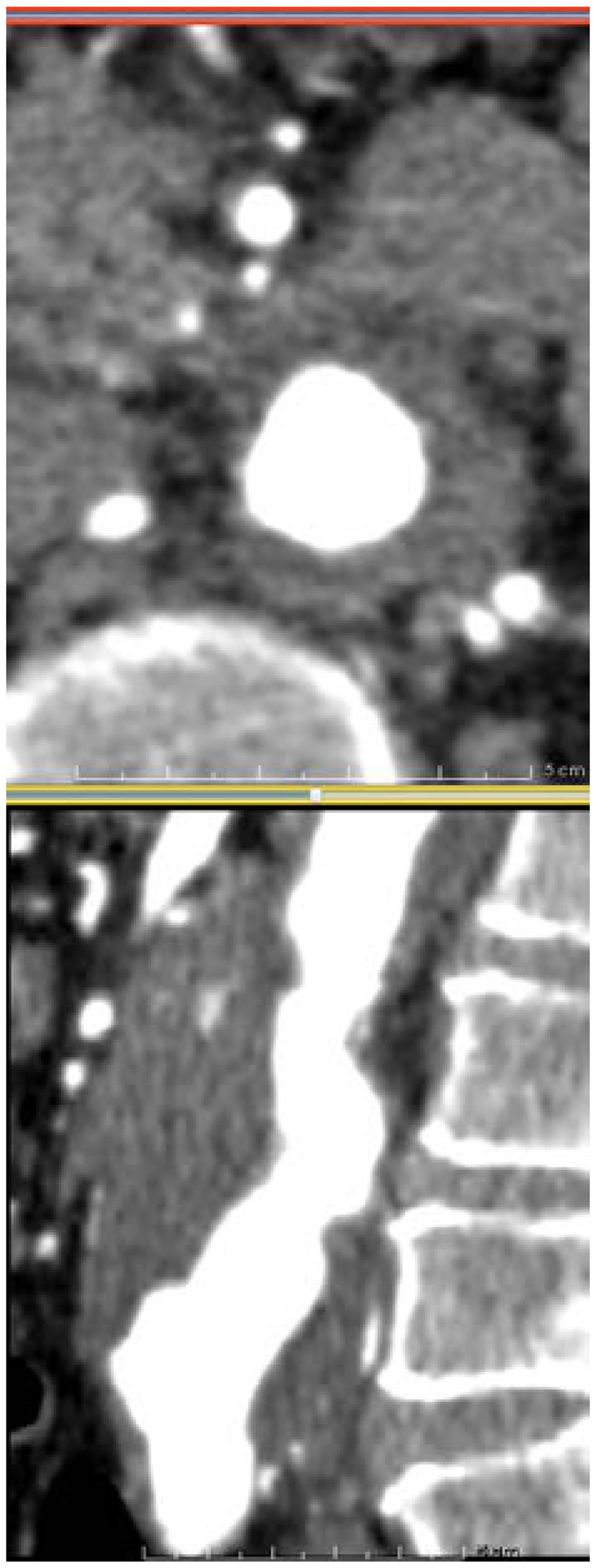
Case 2 used in reproducibility analysis. Axial (top) and sagittal (bottom) CT slices of the AAA. The poor contrast between the AAA and surrounding organs makes segmentation very challenging.

**Figure 4:**
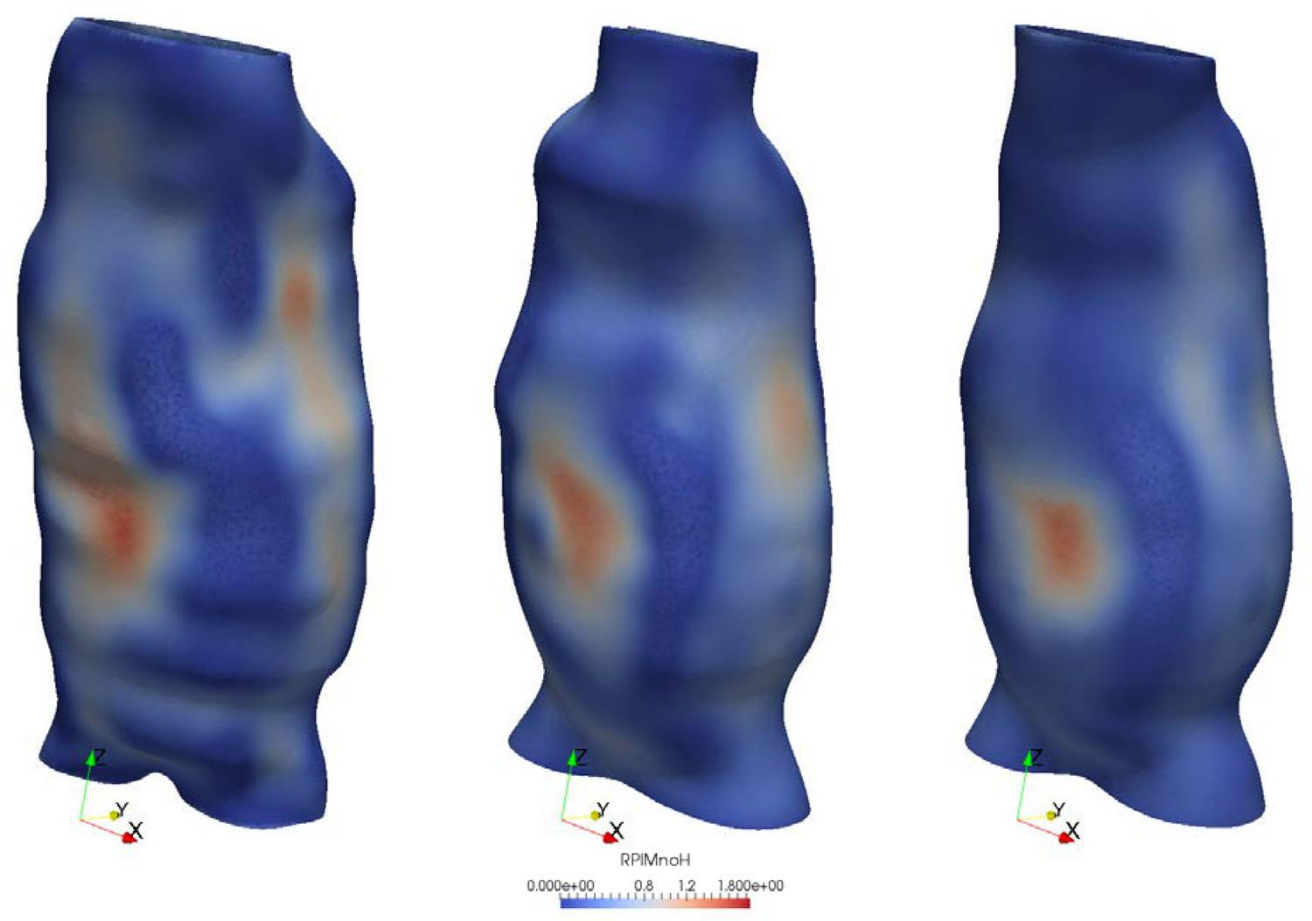
RPI distribution for case 2 used in reproducibility analysis. The results are obtained based on the segmentations performed by user A (right), B (center) and C (left). Despite the visual differences in geometry from user to user, the difference between the maximum RPI is ~5%.

The differences between segmentations must be considered by taking into account the resolution of the CT images, of 0.625 mm × 0.625 mm × 2 mm (therefore, a one voxel segmentation error results in a 2.5% AAA radius error) and the lack of contrast in parts of the image, as illustrated in Fig. 3. However, despite the relatively large difference in peak wall stress calculated between users, the difference between the maximum computed RPI for this case is approximately 5%. This is due to the RPI calculation including information specific to the 3D reconstruction (i.e. NORD and ILT thickness). The computed RPI for this case is presented in Fig. 4.

A more detailed study of inter-user and intra-user reproducibility of CT segmentation and its effect on the computation of wall stress is presented in Hyhlik-Durr *et al*.^22^. In that study the commercial VASCOPS software was used to perform semi-automatic segmentation of CT images having much higher resolution (in plane resolution 0.33 mm, slice thickness 0.7–1.0 mm). The authors reported a high reproducibility of volume and maximum diameter measurements in infrarenal AAAs. We note the fact that segmentations obtained using the VASCOPS software can also be used as inputs for our software system.

### Comparison between different modelling scenarios

We used three different modelling scenarios and assumed the AAA wall was of uniform thickness (1.5 mm) and the ILT was a homogeneous material:

- AAA with ILT and the blood pressure load applied on the ILT surface (ILT pressure);
- AAA with ILT and blood pressure load applied on the internal wall surface, bypassing the ILT (Wall pressure);
- AAA without ILT and blood pressure load applied on the internal AAA wall surface (No ILT).

**Figure 5:**
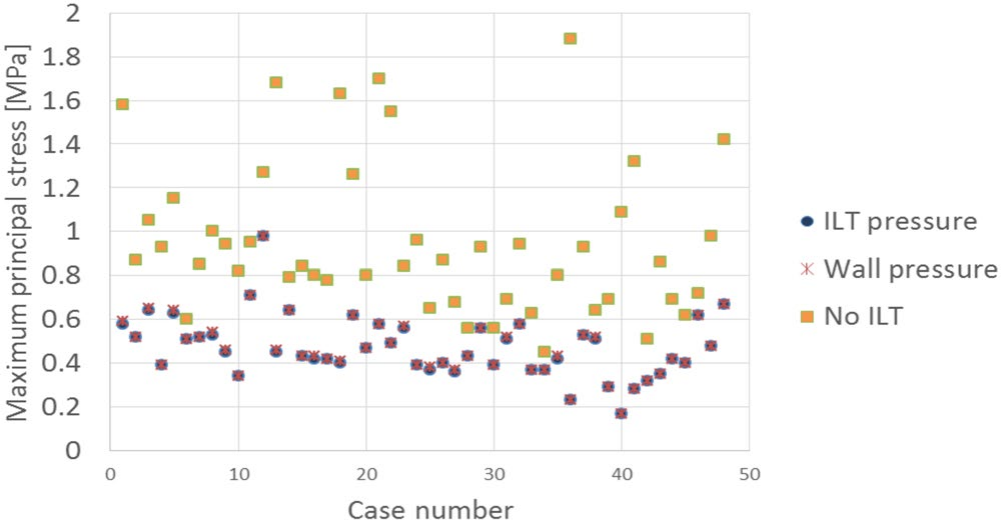
Influence of modelling scenario on the computed stress. Three scenarios are compared. There is little difference between the stresses computed using the “ILT pressure” and “Wall pressure” scenarios. The inclusion of ILT results in a significant reduction in the computed stress.

A comparison between the maximum principal stresses obtained for each case is presented in Fig. 5. While the inclusion of ILT results in a significant reduction in the computed maximum principal stress, there is little difference between the stresses computed using the “ILT pressure” and “Wall pressure” scenarios, as previously reported^27^. The stresses computed using the “Wall pressure” scenario are slightly higher than those computed using the “ILT pressure” scenario, but the maximum difference across all cases is less than 3%. Therefore, there is no need to analyse these two scenarios separately, and only the “ILT pressure” scenario results will be considered in the following analysis.

**Figure 6:**
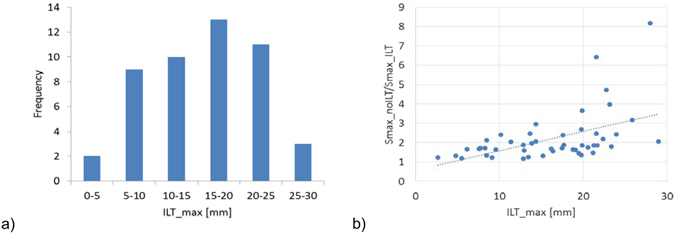
Influence of ILT thickness. (**a**) The distribution of maximum ILT thickness for the analysed cases. (**b**) Influence of maximum ILT thickness on the ratio between the maximum principal stresses computed in the “No ILT” and “ILT pressure” modelling scenarios.

To better understand the influence of ILT on the computed stress, we plotted the reduction in stress between the “No ILT” and “ILT pressure” modelling scenarios against the maximum ILT thickness in Fig. 6. We find that a larger ILT thickness leads to a larger reduction in the maximum principal stress (up to 8 times higher wall stress without ILT). Clearly, the decision on whether or not to include the ILT in the analysis has a major influence on the computation results, especially for cases with large ILT thickness.

### Influence of AAA diameter on stress

**Figure 7:**
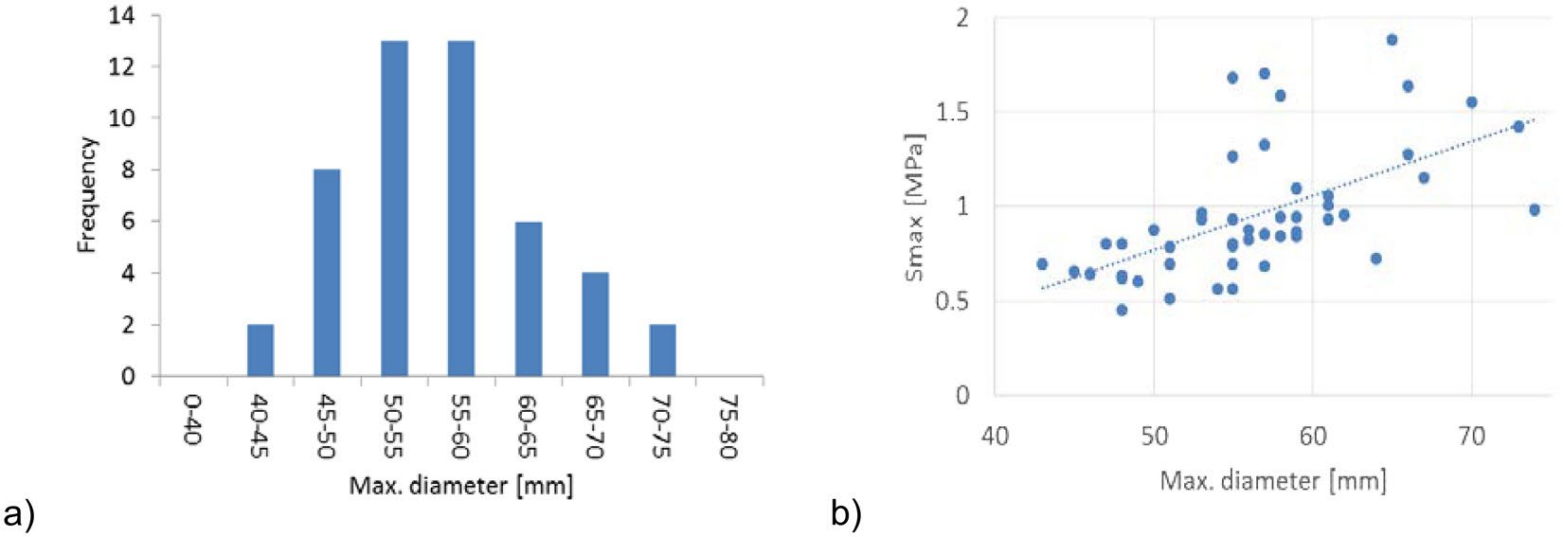
Influence of AAA diameter. (**a**) The distribution of maximum AAA diameter for the analysed cases. (**b**) Variation of computed maximum principal stress with the maximum AAA diameter (R^2^ = 0.34). The “No ILT” scenario results are presented, to eliminate the influence of ILT thickness on the results.

The relation between the maximum AAA diameter and maximum principal stress for all the analysed cases is presented in Fig. 7. Only the “No ILT” scenario results are presented, to eliminate the influence of ILT thickness and only capture the influence of AAA diameter on the computed stress. There is a trend of increased maximum principal stress with increased AAA diameter, although there are other factors influencing the stress value (such as the shape of the AAA) which lead to a relatively large dispersion of the results (R^2^ = 0.34).

### Relation between AAA diameter, ILT, family history of AAA and RPI

**Figure 8:**
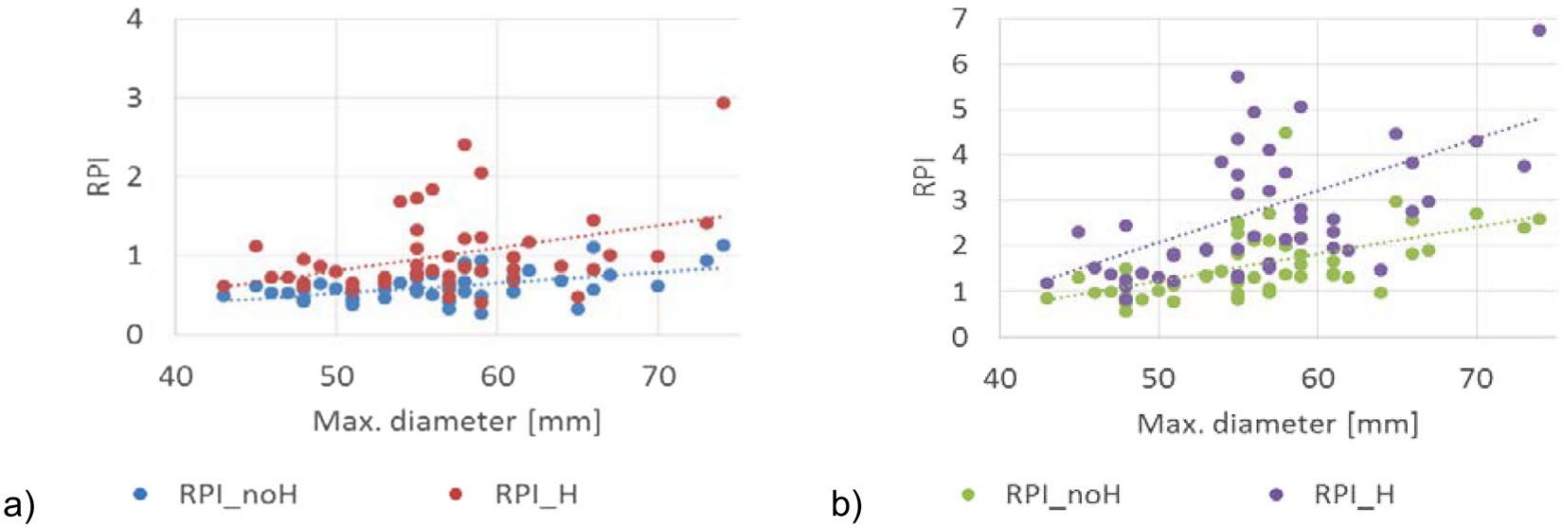
Influence of AAA diameter, ILT, and family history of AAA on the computed RPI. (**a**) RPI computed in the “ILT pressure” scenario, with (R^2^ = 0.16) and without (R^2^ = 0.25) considering a family history of AAA for each case. (**b**) RPI computed in the “No ILT” scenario, with (R^2^ = 0.18) and without (R^2^ = 0.3) considering a family history of AAA for each case.

The variation of computed maximum RPI against the maximum AAA diameter for the “ILT pressure” and “No ILT” scenarios, with and without considering the family history of AAA for each case, is presented in Fig. 8. In each modelling scenario there is a trend of increased RPI with increased AAA diameter, especially when ILT is omitted from the model. Furthermore, without ILT, most RPI exceed the theoretical rupture value of 1.0, yet all AAAs were intact when images were acquired. This further supports the necessity to include ILT into the risk assessment models. We also found that the parameters influencing the wall strength, such as family history of AAA, have a major impact on the RPI computation (the wall strength is computed based on a statistical model^8^, as described in the section “Rupture Potential Index Computation”), reducing the correlation between RPI and maximum diameter, as indicated by the smaller R^2^ values.

In all the presented results, although some trends can be identified, there is a large dispersion of the data. Due to the influence of multiple parameters on the computed stress/RPI, simplistic conclusions such as “larger ILT thickness leads to a smaller stress” or “larger AAA diameter leads to larger stress” cannot be drawn. The obtained results suggest that patient-specific analysis is needed in each case.

## Methods

### Image segmentation

**Figure 9:**
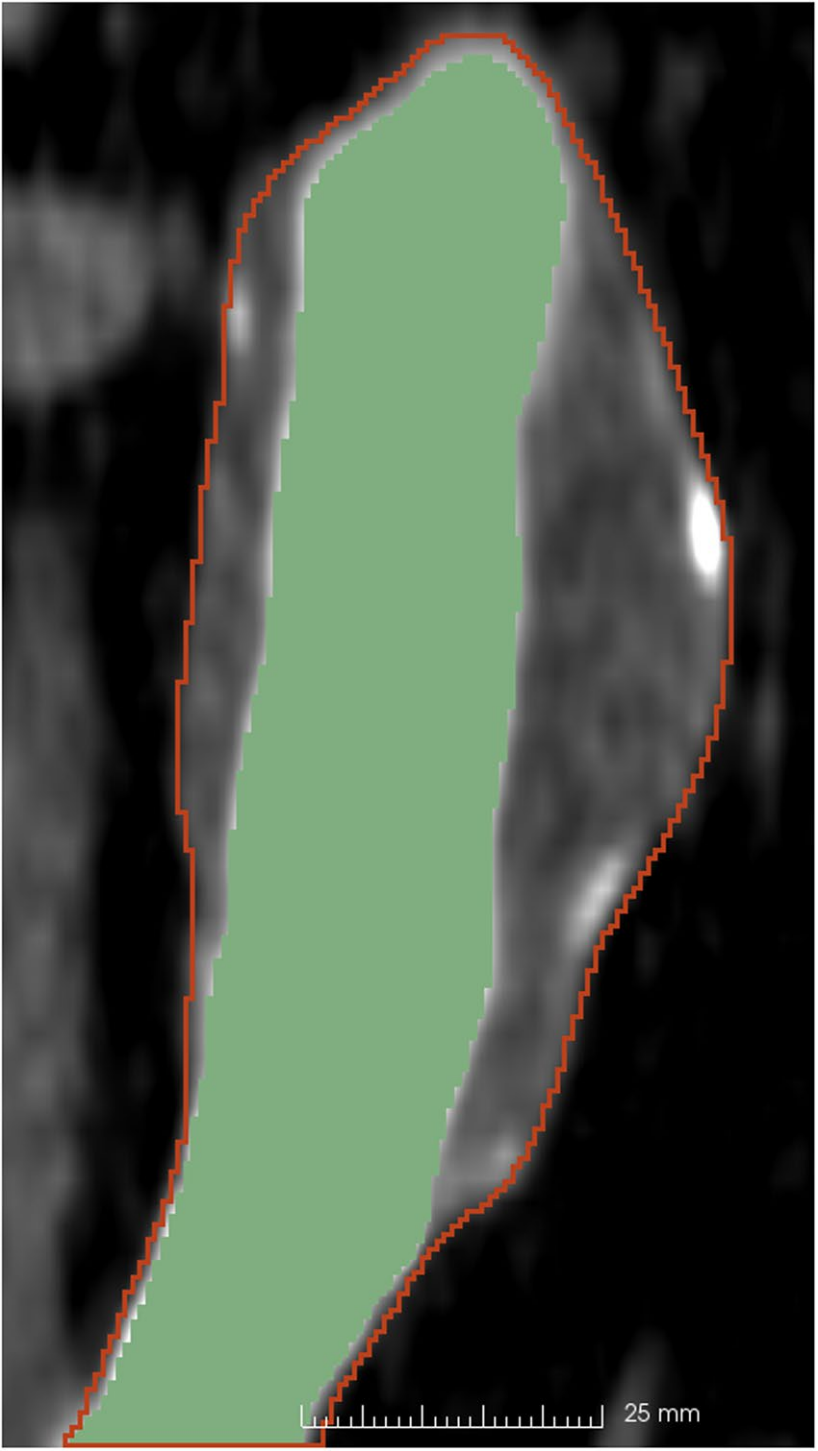
Example segmentation of an AAA from CT (one CT slice in the coronal view). The contour of the AAA segmentation is shown in red and the lumen segmentation is in green. The region of interest has been extracted from an abdominal CT with a resolution of 0.625 mm × 0.625 mm × 2 mm and segmented using the FastGrowCut algorithm for the AAA and intensity thresholding for lumen (with manual seed creation and segmentation corrections).

The high variability in AAA geometry, as well as low discrimination between the AAA and the surrounding tissue in parts of the image, make automatic AAA segmentation practically impossible. Therefore, we have used the segmentation tools available in the free, open source image analysis software 3D Slicer^28^. Any other segmentation software can be used. We have found that using the 3D Slicer extension FastGrowCut for segmentation^29^ can help reduce the segmentation time. Manual intervention is still required in defining the region of interest in the image, cropping, defining the seeds for the FastGrowCut algorithm and performing corrections and smoothing of the resulting label maps. Using this method, we can easily extract the AAA from CT or MRI (Fig. 9). In the preliminary data presented in this work, we have segmented the AAA from immediately below the renal arteries to the iliac bifurcation.

### Inter-modality image registration

**Figure 10:**
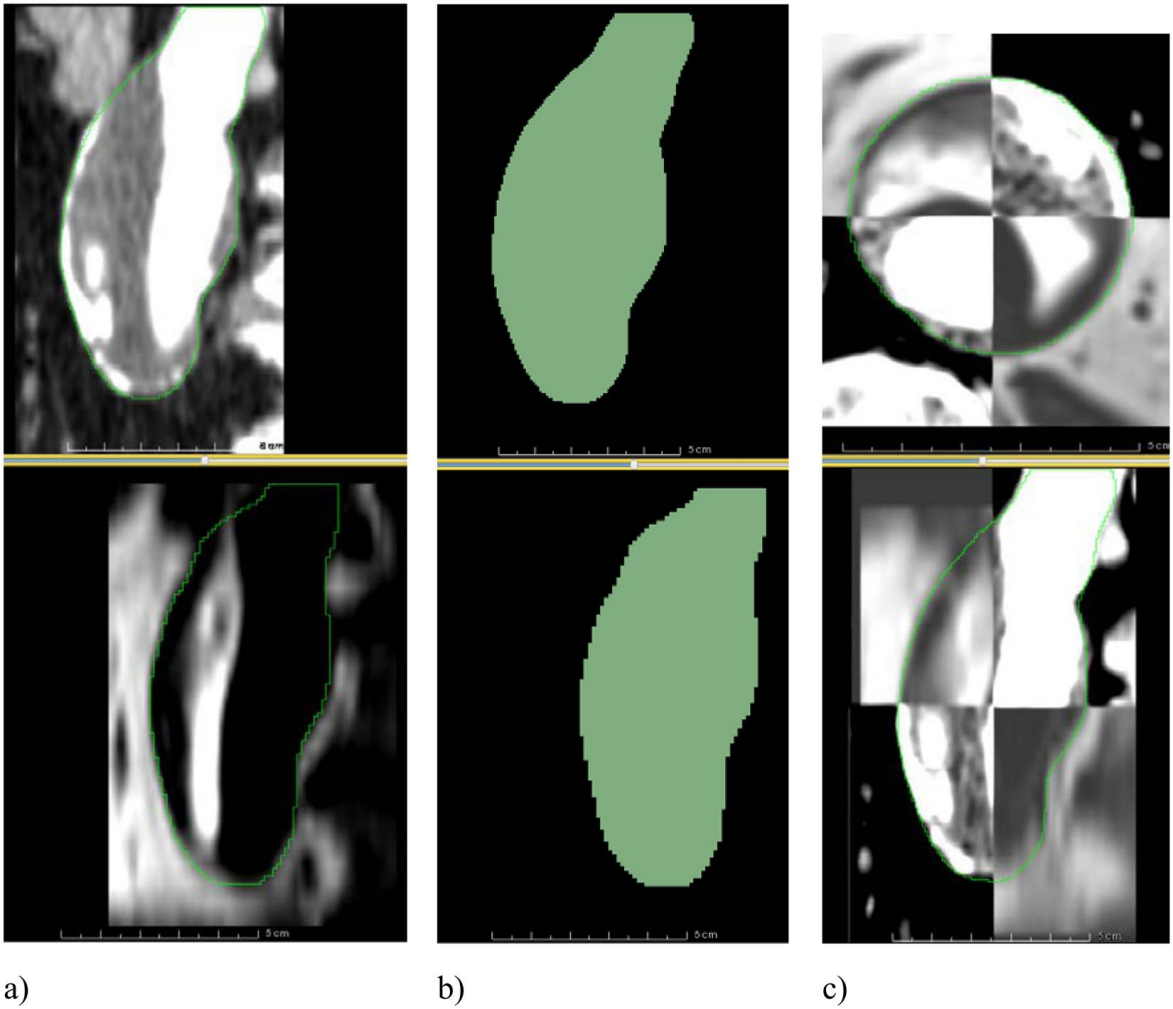
MRI to CT registration. (**a**) A sagittal slice of an AAA in a contrast-enhanced CT image (top) and the corresponding slice in the MR image (bottom). The contours of the AAA segmentations are shown in green. The two images occupy different positions in space and need to be registered. (**b**) The segmentations of the CT (top) and MR (bottom) images. The two label maps are registered and the resulting transform is saved. (**c**) The transform obtained from the label map registration is used to register the MRI dataset to the CT dataset. A checkerboard display of the two images is used to verify the registration result in axial (top) and sagittal (bottom) views, with the contour of the AAA segmentation shown in green.

Registering inter-modality images allows the user to extract geometrical information, such as the AAA wall thickness, from medical images from different imaging systems. Registration involves aligning different images into the same coordinate system. Automatic registration of inter-modality images is practically impossible most of the time, because different image modalities can reveal and represent very different information about an organ. In order to overcome this difficulty, we implemented a label map based registration algorithm and used it to register MRI to CT images. The basic principle of the algorithm is to register the AAA label maps extracted from MRI and CT and then use the obtained transform to bring the MRI image in the same coordinate system as the CT image (Fig. 10), using the following steps:

- Segment the AAA from both CT and MRI (Fig. 10a), resulting in 2 binary images defining the label maps (Fig. 10b);
- Register the MRI label map to the CT label map and extract the resulting transform;
- Use the transform to register the MRI image to the CT image (Fig. 10c).

These steps are performed using the general registration and resampling algorithms (BRAINSFit and BRAINSResample) available in 3D Slicer. The registration algorithm has been implemented in a Python script so it can be run without user intervention. One disadvantage of this registration method is that it cannot take into consideration local deformations; therefore only rigid registration is implemented.

### Wall thickness specification

Although wall thickness has a great influence on the stress distribution within the AAA wall^19^, accurate extraction from medical images remains problematic due to the low image resolution and soft tissue contrast. This uncertainty is why many authors have used constant wall thickness in their analyses. It is nevertheless important to understand the effect of such an assumption on the computed rupture potential index (RPI). Therefore, we offer the possibility of specifying wall thickness at multiple points on the AAA surface.

**Figure 11:**
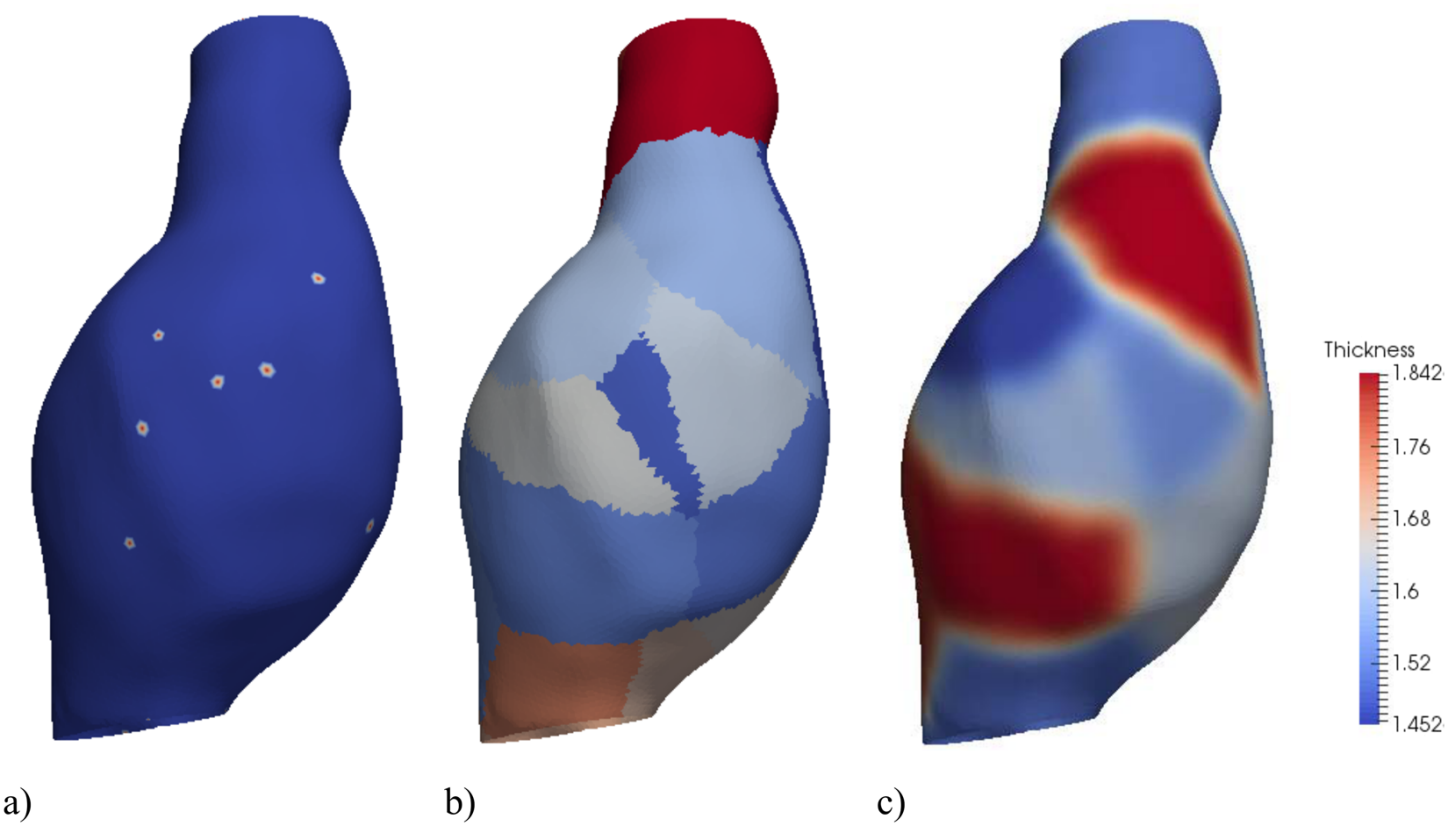
Example of thickness interpolation for an AAA. (**a**) Thickness measurements points. (**b**) Areas of the surface discretization associated with each measurement point (similar to a Voronoi diagram in plane) – the thickness at the measurement point will be extended to these areas. In this image the colors are determined by the measurement point id – for illustration purposes, only a limited number of measurement points are defined in order to have a clear separation of these areas. (**c**) Thickness values (using 10 smoothing iterations) [mm].

Measuring surface thickness from medical images or excised tissue samples is difficult and usually results in measurements only at sparse locations on the AAA surface. Therefore, we devised a new method of generating the thickness information for all points of the AAA surface using interpolation and smoothing of the sparse measurements (Fig. 11).

Thickness information can be specified in different ways. One possibility is to measure the wall thickness at different locations with the ruler tool available in 3D Slicer on the registered images (i.e. CT and MRI), and then save the measurements as 3D Slicer annotation files. The software can read the thickness from these files. Another option is to generate the annotation files (which are simple text files) containing thickness information using some other method. For example, *ex vivo* measurements of excised tissue can be performed^30^ or wall thickness can be estimated from CT^13^ and the thickness data entered into the annotation files. If only one file containing thickness information exists, a constant thickness AAA wall will be created. In the preliminary data presented in this study, we used a constant thickness of 1.5 mm.

### Geometry creation

The label maps segmented from the images and the wall thickness information are used to create the AAA geometry. The following surfaces are automatically created: the external AAA wall surface, the internal AAA wall surface and the internal intraluminal thrombus (ILT) surface. The ILT surface is only created if the lumen label map is available. In few cases of AAA (~5%), there is no ILT present^31^.

**Figure 12:**
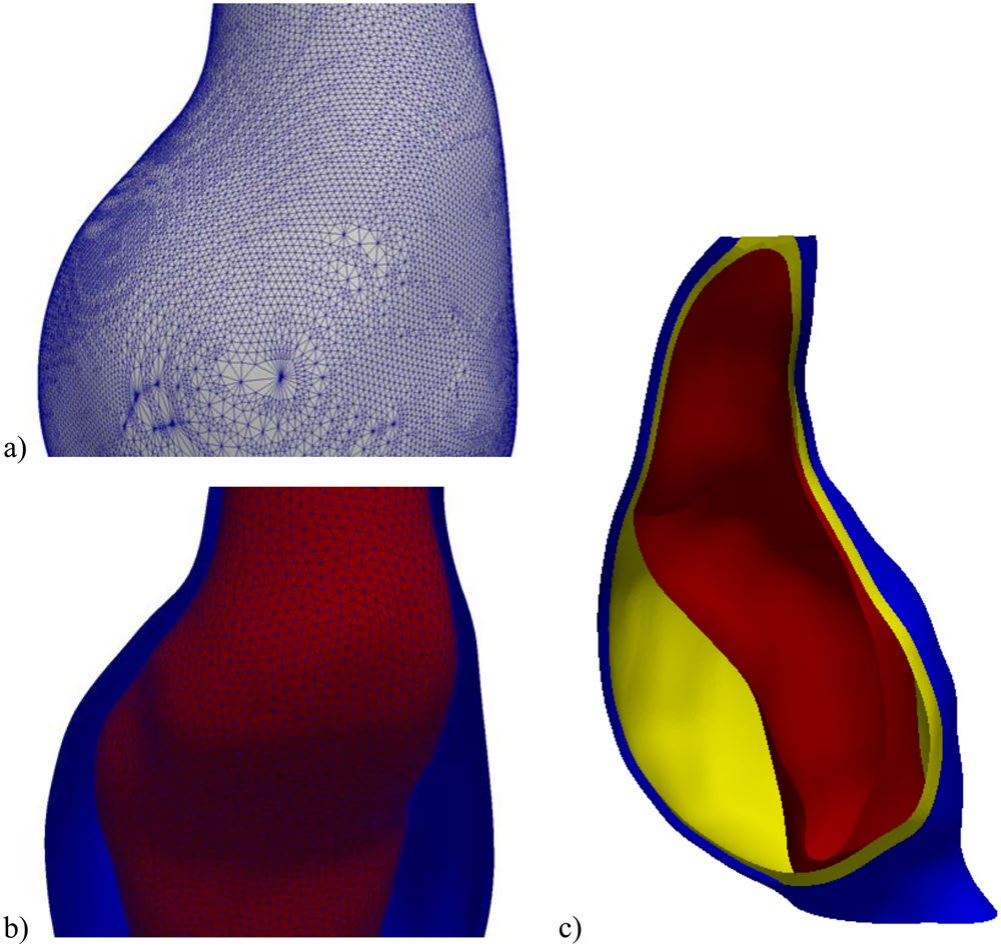
Example of geometry creation. (**a**) Geometry extracted from label map. (**b**) Re-meshed geometry. (**c**) Final geometry (AAA exterior surface in blue, interior surface in yellow and ILT surface in red).

The AAA geometry is created in three stages. The first stage is performed using 3D Slicer and consists of label map manipulation (to make sure the lumen label map is contained within the AAA label map), subtraction of the lumen label map from the AAA label map, and surface extraction using the 3D Slicer module Model Maker^32^. Because the resulting tessellated surface discretisation is only intended for visualization, it has many triangles of different sizes and bad aspect ratios (Fig. 12a). This leads to problems in creating the internal AAA wall surface in the third stage. Therefore, in the second stage, the surface is automatically re-meshed using the surface mesh resampling software ACVDQ^33–35^ (Fig. 12b).

In the third stage, a custom command line interface (CLI) 3D Slicer module is used to generate the discretised surfaces. The module uses the re-meshed AAA surface from the previous stage to separate the exterior AAA wall surface from the ILT surface (if it exists), interpolates the thickness measurements over the external AAA wall surface, creates the internal AAA wall surface by displacing the nodes of the external AAA wall surface along the surface normal, modifies the internal ILT surface to make sure that the ILT has a configured minimum thickness (to simplify ILT meshing, the default value is 1 mm), computes the ILT surface thickness, and outputs the created surfaces (in standard STereoLithography. STL file format) and element size information to be used for meshing.

The element size is computed based on the local wall and ILT thickness, so that a configured number (default value of 2) of element layers are generated over the thickness of the wall. The element size information is generated for all the points of a 1 mm spaced structured grid which covers the entire AAA geometry and saved in a format readable by the meshing software.

### Meshing

**Figure 13:**
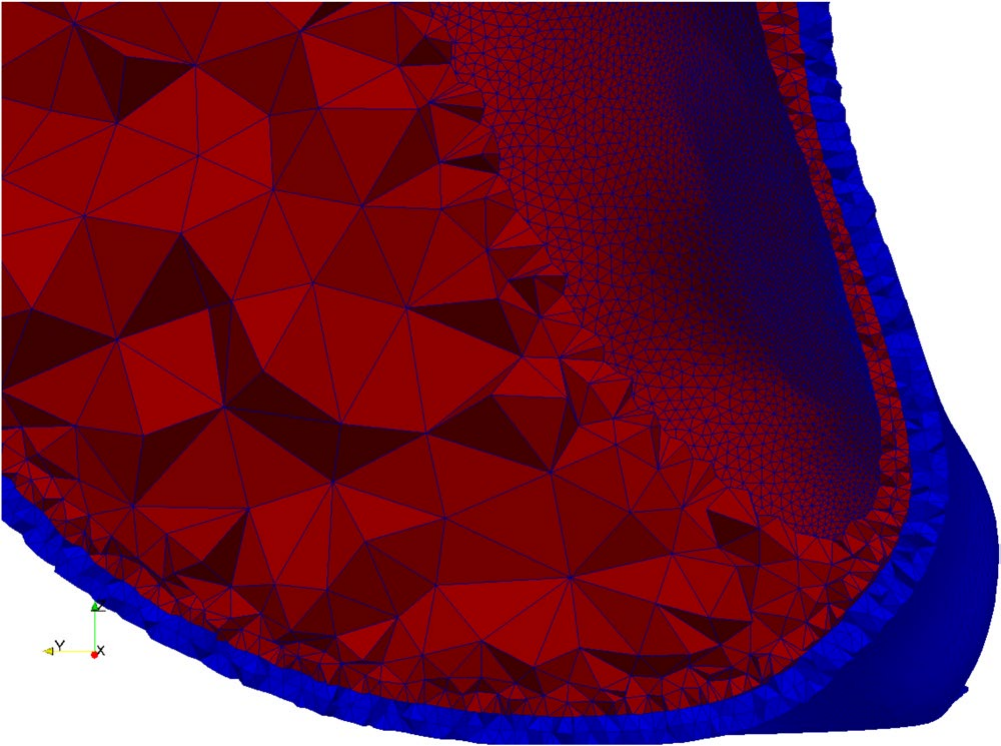
Example of meshing. The AAA wall is meshed using 2 layers of elements (configurable). The ILT is meshed using a minimum of 2 layers of elements (configurable); the element size is increased in the middle of the ILT layer to reduce the number of elements in the mesh.

Meshing of the AAA wall and ILT, based on the surfaces and element size configuration from the previous step, is performed in three stages using custom command files for the free, open source meshing software Gmsh^36, 37^. In the first stage, the surfaces are meshed using the generated element size information. Additional constraints on the element size can be included by modifying the command files, such as curvature dependent element sizing. In the second stage, the volumes of the AAA wall and ILT are created by generating end surfaces between the external and internal AAA wall surfaces, and between the internal AAA wall surface and the ILT surface. We perform this necessary step to ensure the geometry consists of watertight closed surfaces, as this is not guaranteed by the VTK toolkit^38^ used to create surfaces in the previous step. In the third and final stage, a tetrahedral volumetric mesh is created using the element size information generated in the previous step (Fig. 13). This process ensures a conforming mesh between the ILT and AAA wall. The generated meshes are saved as standard.vtk files.

This new meshing approach maintains the geometric accuracy of the meshed surfaces by using very small elements on these surfaces. At the same time, by increasing the element size inside the ILT volume and in the thicker areas of the AAA wall, it reduces the mesh size and, therefore, the computational cost of the finite element analysis.

### Finite Element Model Creation

A custom CLI 3D Slicer module reads the volumetric mesh files created in the previous step and generates input (.inp) files for the finite element software Abaqus^21^ containing the AAA wall and the ILT meshes as parts. The surfaces necessary for defining loads and boundary conditions are automatically detected and included in the generated files and the element type can be configured as linear or quadratic, displacement only or hybrid displacement-pressure formulation. The generated files are then copied into separate folders for each simulation scenario that needs to be analysed.

**Figure 14:**
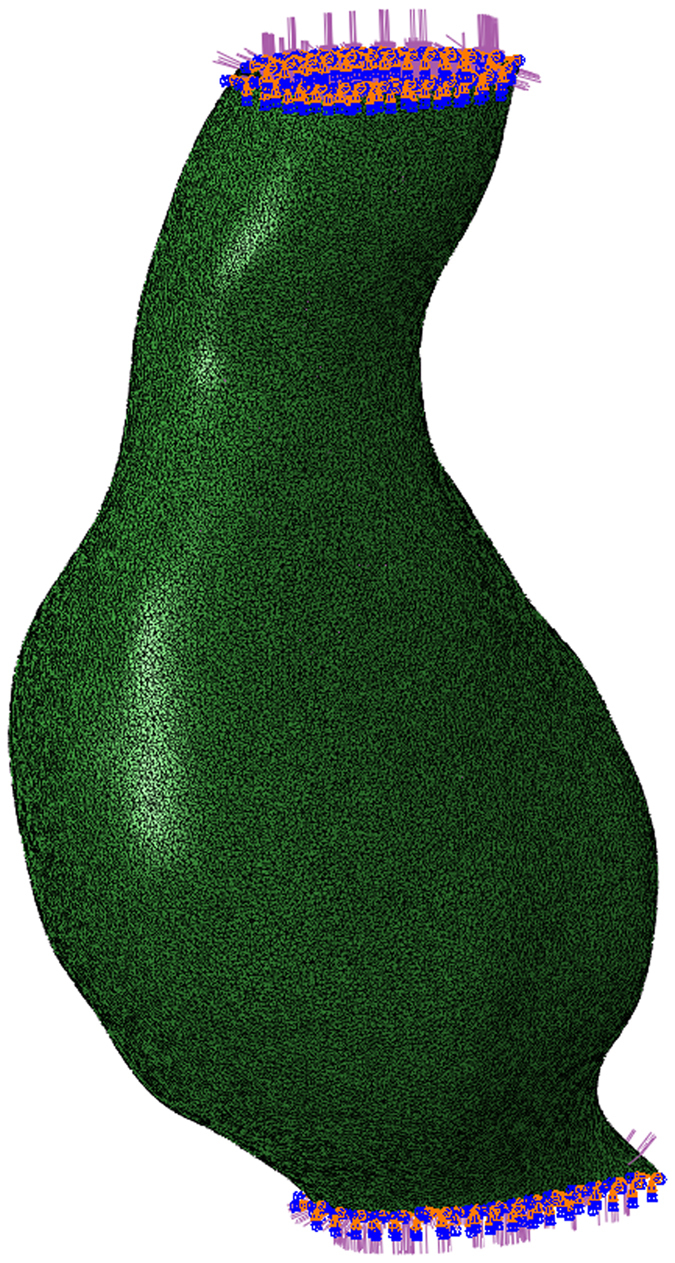
Example of Abaqus model, showing constraints applied on the top and bottom surfaces of the AAA. Definitions of the important surfaces in the model (where loads and boundary conditions are applied) are created by the software and available to the user in the generated input files.

Three analysis scenarios are currently included: (i) AAA with ILT and the blood pressure load applied on the ILT surface; (ii) AAA with ILT and blood pressure load applied on the internal wall surface, bypassing the ILT; and (iii) AAA without ILT and blood pressure load applied on the internal AAA wall surface. These scenarios are a result of uncertainty in the role of ILT. The ILT is believed to buffer the blood pressure being applied to the wall^16^, thus reducing wall stress. However, *in vivo* measurements with a pressure probe placed directly into the ILT showed no reduction in pressure^39^. Therefore, the ILT is thought to act similar to a series of ropes, anchoring the AAA wall but enabling pressure transmission^27, 40^. For each scenario, an Abaqus input file defines the simulation parameters and includes the generated AAA wall and ILT mesh files in order to define the geometry, loading and boundary conditions (Fig. 14). Therefore, the user has complete control over each simulation scenario by simply editing the Abaqus input file corresponding to that scenario. More information on how to edit, add or remove a simulation scenario is included in the configuration instructions accompanying the software.

### Finite Element Analysis

The finite element analysis is performed using the commercial finite element software Abaqus, for each configured simulation scenario. The simulations are carried out using the procedure described in Joldes *et al*.^19^, which allows the computation of stress in the AAA wall without exact knowledge of the material properties. The approach exploits the fact that the geometry of an AAA extracted from medical images represents the deformed geometry (under internal blood pressure). The AAA in the deformed configuration is a statically determined structure. Therefore, the internal stress has to balance the externally applied forces; the stress is determined by the applied load and the AAA geometry and is very weakly dependent on the material properties. This is of great practical significance, as patient-specific material properties for the AAA wall and ILT are difficult to obtain *in vivo*. Removing the dependency on material properties is a major innovation in AAA biomechanics research. For a detailed discussion of the problem of obtaining solutions without knowing mechanical properties of tissues please see also Miller *et al*. and Wittek *et al*.^18, 41^.

**Figure 15:**
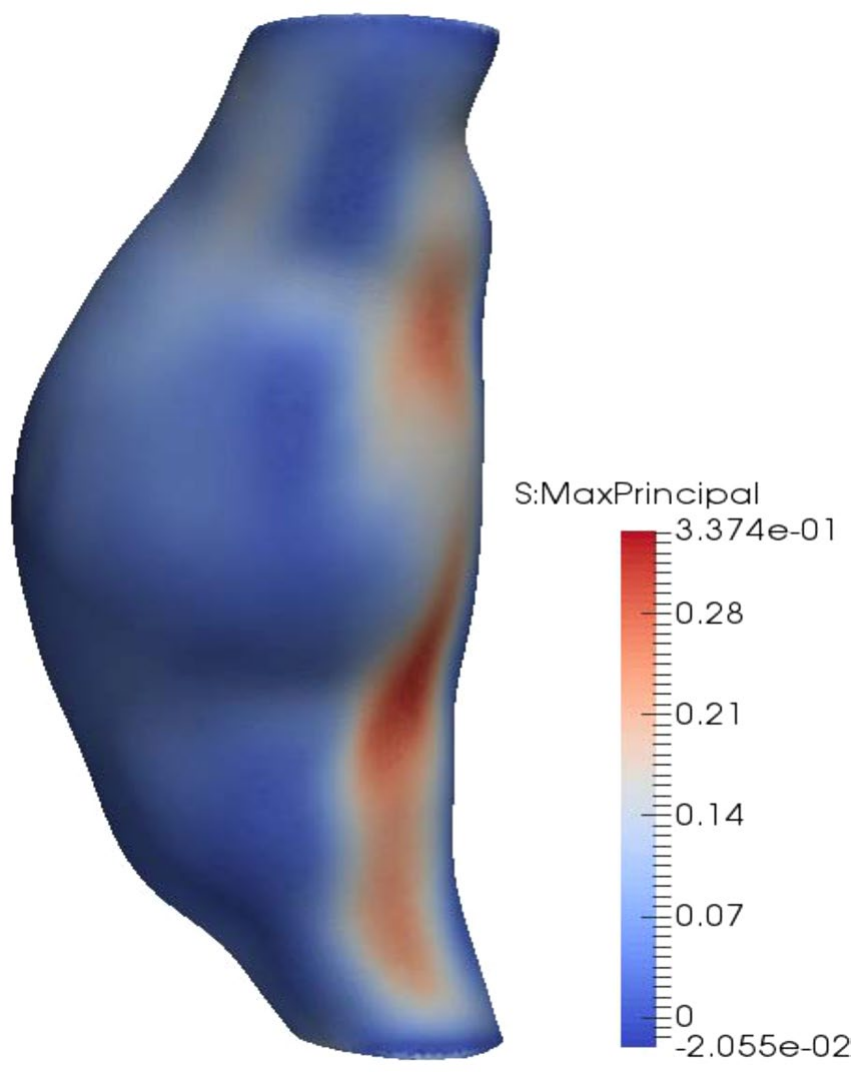
Example of stress computation results. The maximum principal stress [MPa] is computed at each node of the finite element mesh.

The results of the finite element simulation (maximum principal stresses in the AAA wall) are extracted using the Abaqus scripting interface and saved in a.vtk file (Fig. 15).

### Rupture Potential Index Computation

We used the statistical model for AAA wall strength proposed by Vande Geest *et al*.^8^, which has subsequently been used in several AAA rupture risk assessment studies^3, 4, 9–11^. The wall strength at any point of the AAA surface is determined based on the following variables: local ILT thickness (ILT), normalized AAA diameter (NORD), sex of the patient and whether or not the patient has family history of AAA. NORD is the ratio of the maximum AAA diameter to the diameter at the proximal aorta distal to the renal arteries.

By combining the computed wall stress with the wall strength information, the rupture potential index (RPI) can be computed at any point of the AAA surface as the ratio between wall stress and wall strength^2^. A RPI of one is the theoretical point of rupture as the local stress higher has exceeded the local strength; therefore, values above one imply a high likelihood of rupture.

The RPI is computed using a custom CLI 3D Slicer module, and the results (including intermediate variables used for wall strength computation, such as ILT thickness and normalized diameter) are saved in a Visualisation Toolkit Polygonal data (.vtp) file for visualization and assessment (see Fig. 2).
